# UpSETing chromatin during non-coding RNA production

**DOI:** 10.1186/1756-8935-6-16

**Published:** 2013-06-05

**Authors:** Swaminathan Venkatesh, Jerry L Workman, Michaela Smolle

**Affiliations:** 1 Stowers Institute for Medical Research, 1000 E 50th Street, Kansas City, MO 64110, USA

**Keywords:** Chromatin, Nucleosomal organization, Cryptic transcription

## Abstract

The packaging of eukaryotic DNA into nucleosomal arrays permits cells to tightly regulate and fine-tune gene expression. The ordered disassembly and reassembly of these nucleosomes allows RNA polymerase II (RNAPII) conditional access to the underlying DNA sequences. Disruption of nucleosome reassembly following RNAPII passage results in spurious transcription initiation events, leading to the production of non-coding RNA (ncRNA). We review the molecular mechanisms involved in the suppression of these cryptic initiation events and discuss the role played by ncRNAs in regulating gene expression.

## Introduction

**Figure 1:**
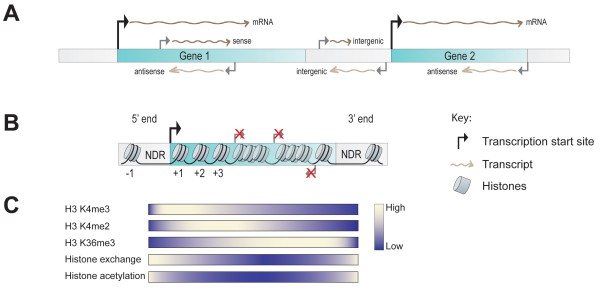
**Non-coding RNAs and chromatin organization. **(**A**) Protein-coding genes are transcribed in the sense direction in order to produce mRNA. Transcription of ncRNAs can be initiated from genic as well as intergenic regions in both the sense and antisense directions. Often transcription is initiated from nucleosome depleted regions (NDRs) in a bidirectional manner. (**B**) Typical nucleosomal organization of yeast genes. NDRs are generally found just up- and downstream from coding regions. A highly positioned +1 nucleosome covering the transcription start site (→) directs precise positioning of nearby nucleosomes, but its influence wanes with increasing distance from the TSS. The presence of nucleosomes over coding regions masks the cryptic promoter elements found throughout the genome and thus prevents aberrant transcription initiation. (**C**) Transcription-dependent distribution of H3 K4 and H3 K36 methylation over gene coding regions. Heatmaps also show high levels of histone acetylation and exchange over promoter-proximal regions which anticorrelate with the distribution of H3 K36 trimethylation.

Transcription of genomic regions in eukaryotes is a complex phenomenon generating a variety of RNAs, only a subset of which is derived from protein coding genes (mRNAs). The non-coding transcriptome includes numerous RNA species involved in the regulation of translation (tRNAs and rRNAs), but more recent studies have indicated the presence of several types of RNA molecules that have the potential to regulate gene expression [1,2]. Newly developed techniques employed in the analysis of the eukaryotic transcriptome such as RNA-Seq (reviewed in [1]) suggested widespread transcription of up to 85% of the yeast genome [3] and 75% of the human genome [4]. Apart from transcription of coding regions, pervasive transcription results from the use of multiple transcription start sites (TSS) [5], resulting in overlapping transcripts (Figure 1A). Alternatively, transcription may be initiated from the ends of genes, thus giving rise to antisense [6] and intergenic transcripts [7] (Figure 1A). In addition, start sites hidden within the transcribed region of genes are accessed by the RNAPII under certain conditions, resulting in cryptic transcription in both the sense and antisense directions (Figure 1A) [8-10]. One characteristic feature of pervasive transcription is its tight regulation. Expression of ncRNAs is observed only in particular growth conditions, and usually to a lower extent when compared to the mRNA levels of protein-coding genes [4,11,12], suggesting a regulatory role for these molecules.

Eukaryotic organisms use the ordered packaging of genomes into chromatin as a means of regulating gene expression (reviewed in [13,14]). Indeed, nucleosome occupancy across transcription regulatory regions in yeast is indicative of the rate of transcription from coding regions [15,16]. In addition, several transcription elongation factors that have been found to control transcriptional output from a given gene, have also been shown to possess the ability to either directly or indirectly influence nucleosome stability [8,17-19]. Consequently, a number of these elongation factors also play a role in the regulation of cryptic transcription [20,21]. We discuss how the maintenance of the underlying chromatin structure is necessary for the controlled expression of non-coding RNA molecules.

Finally, the stability of RNA molecules is a consequence of the balance between transcriptional output and the degradation mechanisms that ensure RNA destruction. Recent publications have investigated different RNA degradation pathways that play a role in the production of ncRNA and provide one means of classifying different types of ncRNAs [7,22-24].

## Review

### Chromatin organization and transcription initiation

Nucleosomal stability varies based on the genomic location, and cells exploit these differences to regulate DNA based processes. Access to nucleosomal DNA is also facilitated by the action of chromatin remodeling factors, which are in turn aided by post-translational modifications present on histones [25]. These histone modifications possess the additional function of either recruiting or repelling nucleosome regulatory factors, thereby affecting the functional outcome. We shall briefly review the distribution of nucleosome organization in relation to the gene structure and its influence on gene expression.

Technological advances in the field of genomics (ChIP-chip and ChIP-Seq) merged with well-established biochemical tools (MNase digestion) have generated precise nucleosome maps in several organisms [26-32]. Despite variations in the nucleosomal spacing among different cells and organisms, the organization of nucleosomes over genes showed robust similarities. For the purposes of this review, we refer to data obtained from the genome-wide nucleosomal mapping in *S. cerevisiae* as an example of this organization [6,16,26,27,33].

A vast majority of nucleosomes in yeast (ca. 80%) are highly positioned, suggesting that there is very little variability in these positions in a cell population [34]. The most strongly positioned nucleosome is found covering the transcription start site (TSS) and is denoted as the +1 nucleosome (Figure 1B). While the +1 nucleosome is maintained across different species, its position relative to the TSS varies [35]. This nucleosome has been suggested to function as a ‘barrier’, resulting in the ‘statistical positioning’ of nucleosomes downstream [15,34,36]. Positioning of nucleosomes decreases with increasing distance downstream of the ‘barrier’ nucleosome, becoming more delocalized towards the 3’ ends of genes (Figure 1B). Upstream of the TSS and the +1 nucleosome lies the nucleosome depleted region (5’ NDR) (Figure 1B). This region is enriched for poly (dA:dT) tracks, which disfavor nucleosome formation due to the inability of these sequences to bend [34,37]. NDRs are also enriched for regulatory DNA sequences including transcription factor binding sites. Providing an upstream boundary to the 5’ NDR is another positioned nucleosome (-1 nucleosome), the stability and position of which determines access to the regulatory sites in the 5’ NDR (Figure 1B) [37]. Thus, in the event of transcription initiation, this nucleosome undergoes a variety of post-translational modifications and is the target of nucleosome remodelers. The 3’ ends of genes also possess a NDR (3’ NDR) which overlaps with the transcription termination site (Figure 1B).

Transcription initiation usually occurs from the NDRs at both ends of the genes (Figure 1A). Apart from protein coding genes that are transcribed from the promoter, 5’ NDRs may also give rise to intergenic transcripts leading away from coding regions [7,23]. Similarly, intergenic transcripts also arise from 3’ NDRs in addition to antisense transcripts that traverse the gene coding regions [6]. This observation indicated that all nucleosome-depleted regions may inherently function in a bi-directional manner [7,23]. Yet, over a majority of promoters transcription occurs predominantly in one direction only [38].

Gene looping between the promoter and terminator regions is one way to ensure directionality. Association of the polyadenylation complex factor Ssu72 with both the 5’ and 3’ ends of genes mediates gene looping and results in the reengagement of RNAPII, thereby ensuring directional expression of mRNAs. In contrast, loss of *SSU72* leads to increased levels of divergent ncRNA [39].

One of the factors regulating transcription initiation from NDRs is the chromatin remodeler Imitation switch 2 (Isw2) that mobilizes nucleosomes to reduce NDR size [6,38]. Loss of Isw2 leads to reduced nucleosome occupancy over NDRs and the production of ncRNA, often initiated from 3’ NDRs and is mostly transcribed in the antisense direction of known coding sequences [6,38]. An opposing function is carried out by the Remodels Structure of Chromatin (RSC) complex at the 5’ ends of genes [40,41], which maintains an open NDR structure.

The maintenance of chromatin organization throughout the genome is therefore key to preventing aberrant transcription initiation. The cell engages different co-transcriptional mechanisms to maintain chromatin integrity over transcribed genes. In the following sections, we shall discuss the details of these mechanisms.

### Post-transcriptional maintenance of chromatin organization

The nucleosome serves as a strong impediment to RNAPII progression during transcription elongation. Passage of elongating RNAPII through a nucleosome *in vitro* may occur upon loss of a single histone H2A-H2B dimer, leaving a hexameric nucleosomal complex behind [42]. In conjunction with this observation, *in vivo* studies have shown a continuous exchange of the H2A-H2B dimers over the coding regions [43]. However, highly transcribed genes with increased levels of RNAPII over coding regions demonstrate a complete loss of nucleosomes, including H3-H4 tetramers [44]. This suggests that nucleosomal dynamics during transcription elongation are a consequence of RNAPII passage [45]. Conversely, shutting off gene expression results in the reassembly of nucleosomes over gene bodies [46,47]. The prevention of spurious transcription initiation has been attributed to the tight regulation of nucleosomal dynamics over coding regions (Figure 1B) [13,14].

RNAPII employs several protein complexes that aid transcription in a stage-specific manner [48]. Reversible phosphorylation of a key structural feature of RNAPII, the C-terminal domain (CTD) heptapeptide repeats of Rpb1 regulates these dynamic associations [49]. Some of these RNAPII and CTD-associated proteins are histone chaperones that serve to reassemble nucleosomes after passage of the polymerase. In addition, several histone lysine deacetylases (KDACs) are targeted to coding regions by histone methylation and act to prevent the accumulation of histone acetylation, thought to increase chromatin accessibility. In the subsequent section we discuss the different strategies used by the transcriptional machinery for the maintenance of organized chromatin structure following transcription, thereby preventing cryptic transcription initiation.

### Histone methylation and post-transcriptional chromatin maintenance: Set2/Rpd3S pathway

Phosphorylation of the Ser2 residue in the CTD heptad repeats by yeast Ctk1 a few hundred base pairs from the start site to the 3’ end of genes recruits the Set2 lysine methyltransferase (KMT) through its Set2-Rpb1 interaction (SRI) domain [50]. Set2 targets the K36 residue on histone H3 (H3 K36) for methylation, and is responsible for the addition of multiple methyl groups (mono-, di- and trimethylation). Depending on the transcriptional status of a gene and the association of different regulatory proteins with Set2, H3 K36 is methylated in an ordered fashion, with H3 K36 monomethylation towards the 5’ end and trimethylation towards the 3’ end of the coding regions [51] (Figure 1C). Thus, H3 K36 methylation is a co-transcriptional histone modification enriched over the coding region of transcribed genes. What functional role does this graded distribution of H3K36 methylation play in the regulation of co-transcriptional nucleosomal dynamics?

**Figure 2:**
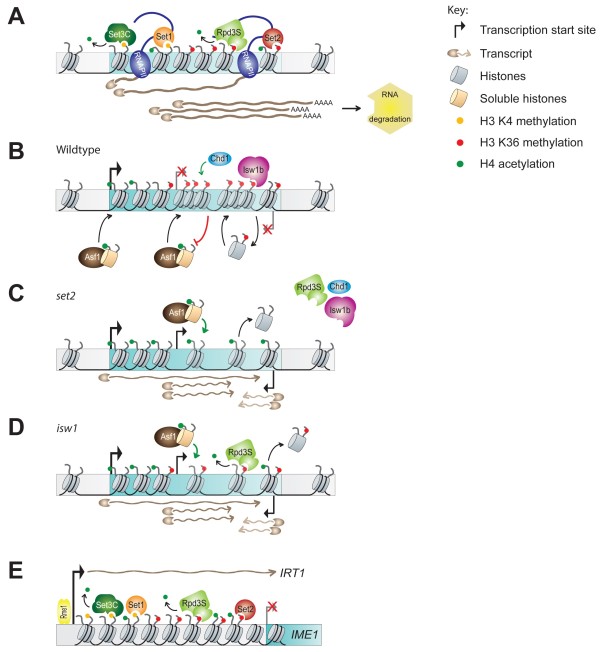
**Organized chromatin structure antagonizes production of ncRNAs. **(**A**) The RNAPII-associated KMTases Set1 and Set2 are required to methylate histone H3 on residues K4 and K36, respectively. H3 K4 dimethylation and H3 K36 trimethylation are essential for maintaining coding regions in a hypoacetylated state: H3 K4me2 directly recruits the histone deacetylase complex Set3C, while H3 K36 methylation is essential for the catalytic activity of the Rpd3S deacetylase complex. (**B**) H3 K36 methylation directly recruits the Isw1b remodeling complex through a PWWP domain in its Ioc4 subunit. Isw1b together with Chd1 are necessary for the retention of existing, H3 K36 methylated nucleosomes over coding regions. Thereby they prevent deposition of soluble, highly acetylated histones through histone chaperones such as Asf1. (**C**) Loss of *SET2* completely abolishes H3 K36 methylation in yeast. Isw1b is no longer correctly recruited to chromatin, resulting in increased histone exchange with a concomitant rise in histone acetylation over coding sequences. This leads to the exposure of cryptic promoters and the production of ncRNAs. (**D**) Loss of either *ISW1* and/or *CHD1* also results in increased histone exchange and histone acetylation even though there is little change in H3 K36me3 levels over the gene body. However, in the absence of the remodeler(s) existing nucleosomes cannot be retained. Instead they are replaced with soluble, highly acetylated histones. Again this process leads to the exposure of cryptic promoters and the production of ncRNAs. (**E**) In haploid yeast Rme1-dependent transcription of the long ncRNA *IRT1* establishes a gradient of H3 K4me2 and H3 K36me2/3 over the promoter of the *IME1* gene. These methylation marks subsequently recruit histone deacetylases Set3C and Rpd3S, respectively, that help to establish a repressive chromatin conformation and thus preclude transcription of *IME1.*

A key observation upon loss of Set2-mediated H3 K36 methylation in yeast is the hyperacetylation of histones over transcribed regions, particularly towards the 3’ end of genes (Figure 1C). Deletion of *EAF3* or *RCO1*, two components of the Rpd3S histone deacetylase complex, also results in a similar phenotype [9,52]. The Rpd3S complex interacts with Ser5 and Ser2 diphosphorylated RNAPII and is thus recruited to the coding region (Figure 2A) [53,54]. Interestingly, the chromodomain-containing Eaf3 subunit binds to methylated H3 K36 and H3 K4 [9,55]. Additionally, the Rco1 subunit mediates the methylation-independent binding of the Rpd3S complex to nucleosomes through a Plant-Homeodomain (PHD) domain. This interaction enhances Eaf3 binding specificity for di- or trimethylated H3 K36 [56,57], thereby stimulating Rpd3-mediated deacetylation of histones H3 and H4 (Figure 2A). Thus, Set2-mediated H3 K36 methylation ensures that transcribed regions remain hypoacetylated (Figure 1C) by coordinating the recruitment and activation of the Rpd3S complex (Figure 2A). Given the role of histone acetylation in promoter chromatin remodeling, leading to transcription initiation, it can be concluded that the Set2/Rpd3S pathway ensures post-transcriptional chromatin integrity by maintaining nucleosomes over the coding regions in a hypoacetylated state.

Evaluation of the nucleosomal dynamics over transcribed regions with respect to the distribution of Set2-mediated H3 K36 methylation, suggested a direct role of the methyl mark in this process (Figure 2B). In yeast, histone exchange over transcribed regions occurs infrequently for a majority of genes, except over highly transcribed genes [58-60]. Loss of H3 K36 methylation resulted in the replacement of histone H3 with H3 from the soluble pool over coding regions, irrespective of the rate of transcription [61] (Figure 2C). Using a different approach, Lieb and coworkers showed that H3 depletion resulted in the loss of nucleosomes over the promoter but not over coding regions which remained enriched for H3 K36 methylated nucleosomes [62]. These results suggest that the H3 K36 methyl mark is required for nucleosomal retention during transcription elongation (Figure 2B). Indeed, the targeting of Set2 to the promoters of active genes has been previously shown to cause transcriptional repression [63]. Interestingly, perturbing the rate of histone exchange in a *SET2* deletion mutant significantly increased the enrichment of preacetylated histones over the coding region (Figure 2C). This indicates that co-transcriptional acetylation is partly a consequence of histone exchange (Figure 2C), in addition to the recruitment of histone acetyltransferase complexes [64]. This result raises the interesting notion that promoter histone acetylation may also be a consequence of histone exchange (Figure 2B).

Loss of the histone chaperone Asf1 results in reduced histone exchange over promoters [19,59], and affects histone exchange over coding regions [65]. Interestingly, H3 K36 di- and trimethylation prevent the interaction of Asf1 with histones over coding regions [61], indicating that the H3 K36 methyl mark may prevent histone exchange by interfering with the binding of histone chaperones involved in post-transcriptional chromatin reassembly [61,66]. Interestingly, Spt6, a histone chaperone involved in post-elongation nucleosomal reassembly is necessary for H3 K36 trimethylation [67,68], indicating that the addition of this mark occurs in conjunction with nucleosomal reassembly following the passage of RNAPII. A recent report further suggests that in human cells, SETD2 is required for the recruitment of the Spt16-containing FACT histone chaperone complex [10]. While the FACT complex demonstrates weaker binding to a H3K36 trimethylated histone peptide [61], it maintained regular binding to the modified nucleosome [69], possibly through known binding interactions with histones and DNA [70]. These studies suggest that the Set2-dependent H3 K36 trimethylation may enhance the functional interactions of FACT to the nucleosome by reducing non-specific charge-based associations, resulting in the efficient removal or exchange of the H2A-H2B dimer while leaving the H3-H4 tetramer intact [10,43]. A similar histone modification-based nucleosome reassembly pathway involving H2B monoubiquitylation and the histone chaperone Spt16 (a component of FACT) has been described for highly transcribed genes in yeast [46,71]. Failure to remove H2B monoubiquitylation over promoters prevented transcription initiation [71,72], in a manner similar to Set2 [63].

Set2-mediated H3K36 methylation also recruits the chromatin remodeling complex Imitation switch 1b (Isw1b) to the coding regions through the PWWP domain of its Ioc4 subunit (Figure 2B) [69,73]. Together with another remodeler, Chromodomain-helicase-DNA binding-1 (Chd1), Isw1b is necessary for the maintenance of chromatin integrity following RNAPII transcription by ensuring the retention of existing, H3 K36-methylated nucleosomes (Figure 2B). Loss of either one or both remodeling enzymes results in increased histone exchange over coding regions as well as increased histone acetylation (Figure 2D) [69,74]. Loss of Isw1 or Chd1 had little effect on H3 K36 methylation levels *per se*, showing that histones were still methylated by Set2 [69,74], but they were no longer retained over coding regions, leading to the exposure of cryptic promoters and increased ncRNA production [69,75,76].

These mechanisms maintain an intact H3-H4 tetramer core, methylated at H3 K36, while H2A-H2B dimer exchange occurs unhindered [43]. Interestingly, this suggests that the H3 K36 methyl mark ensures its persistence over coding regions following transcription elongation. Recently, the core of the Rpd3S complex consisting of its Rpd3, Sin3 and Ume1 subunits [9], was demonstrated to possess a histone modification-independent histone chaperone activity [77]. While the core subunits prevented nucleosome eviction, it did not impede nucleosome remodeling by the RSC complex [77], indicating the possible involvement of another H3 K36me-recruited complex in chromatin reassembly following the passage of RNAPII.

In summary, the H3 K36 methylation mark prevents histone exchange, incorporation of acetylated histones and brings in chromatin remodeling complexes to maintain a spaced chromatin structure, thereby preventing the exposure of cryptic promoter sites over transcribed regions (Figure 2B) [61,69,78]. Indeed, loss of Set2, Ctk1, Rpd3S components, Isw1 and a number of histone chaperones results in the initiation of cryptic transcription [79].

### Histone methylation and post-transcriptional chromatin maintenance: Set1/Set3C pathway

Linking co-transcriptional H3 K36 methylation to the activation of histone deacetylase complex ensures that the 3’ ends of coding regions are hypoacetylated after RNAPII passage. This raises the question of what happens at the 5’ends of genes?

An analogous mechanism has been reported to operate at the 5’ ends of genes. Set1 is recruited to the Ser5-phosphorylated form of RNAPII and specifically mono-, di- and trimethylates K4 on histone H3. While H3 K4 trimethylation is associated with transcriptional activation, Set1-mediated H3 K4 dimethylation recruits the Set3 deacetylase complex (Set3C) through a PHD finger present within the Set3 subunit. Recruitment of Set3C subsequently results in the deacetylation of nucleosomes over the 5’ coding regions by its Hos2 and Hst1 deacetylase subunits (Figure 2A). This mechanism again prevents access to underlying cryptic promoter sequences and initiation from these sites [5,80], although its role in the regulation of histone exchange over the 5’ end of the coding region is as yet unknown.

### What is the role of ncRNAs?

Clearly cells invest a lot of effort to keep ncRNA expression in check. This suggests that some ncRNAs may serve as regulatory molecules under specific growth conditions. Indeed, regulatory roles for a number of ncRNAs have been identified and act through several different mechanisms. Most fundamentally, ncRNAs can act either *in cis* to regulate the genes in the immediate vicinity of its synthesis and/or *in trans* whereby ncRNA molecules affect genes on other chromosomes [81]. *Trans* regulation is rare in yeast where most regulatory ncRNAs identified act *in cis*. However, *trans-*acting ncRNAs are of particular importance for (diploid) higher eukaryotes*.* Examples of regulatory ncRNAs in yeast include *PHO84*[82,83] as well as *SRG1-SER3*[84,85], *IME1*[86], *IME4*[87,88]*, FLO11*[88] and *GAL1*[89,90].

The *PHO84* antisense transcript is unusual for yeast as it affects sense transcription of the *PHO84* gene both *in cis* as well as *in trans* using two different mechanisms [82,83]. Silencing of *PHO84* expression *in trans* requires increased production of its antisense transcript, which in turn is dependent on Set1-mediated H3 K4 trimethylation levels [83]. In contrast, regulation of *PHO84 in cis* depends on another mechanism of ncRNA-mediated repression and involves histone deacetylation by KDAC Hda1 in response to antisense transcription [82,83]. Histone lysine deacetylases are associated with repressing gene expression generally. Another example of KDAC-mediated repression is represented by *IME1*. Meiosis in yeast is a tightly regulated developmental program that occurs in diploid cells alone. The control of the sporulation transcription program rests with the transcription factor Ime1. Upon sensing starvation, diploid yeast cells begin transcribing *IME1*. However, in haploid yeast cells *IME1* is repressed by the Rme1 transcription factor, such that starvation conditions do not result in a lethal cell division. van Werven *et al.* identified a stable unannotated transcript (SUT), *IRT1* (*IME1* regulatory transcript 1) that is regulated by Rme1 (Figure 2E) [86]. The *IRT1* transcript abolished the NDR over the *IME1* promoter by recruiting the Set3 and the Rpd3S deacetylase complexes. This recruitment occurred through the respective methyl marks recognized by these deacetylase complexes (Figure 2E). Loss of both Set2 and Set3 activated the *IME1* gene, despite the continued transcription of the *IRT1* ncRNA, suggesting that both these proteins are important for repression [86]. Repression of *GAL1-10*[89] genes also involves the recruitment of deacetylase complexes.

Another mechanism involving ncRNA-mediated gene repression is exemplified by transcriptional interference as observed for the *SRG1-SER3* gene pair. *SRG1* is a short ncRNA that is transcribed upstream of the *SER3* promoter. *SRG1* transcription positions nucleosomes over the *SER3* promoter, preventing the binding of transcription factors and thereby suppressing its expression [84,85].

## Conclusions

Transcription of non-coding RNAs is a tightly regulated process. Recent studies have identified some of the underlying mechanisms involved that rely on maintaining highly organized chromatin structure throughout transcription. This results in adequate masking of potential cryptic promoters by nucleosomes and thus ensures that they are not available for recruitment of the transcriptional machinery. Perturbations of this system by affecting nucleosome stability, occupancy or histone dynamics all result in wide-spread spurious transcription.

While this review focuses on mechanisms regulating non-coding transcription in yeast, many of the fundamental mechanisms are also expected to apply to higher eukaryotes. Pervasive transcription of ncRNAs has been identified in higher eukaryotes where they act both as repressors as well as activators of gene expression and perform such regulatory roles *in cis* as well as *in trans*[2]*.*

*Cis-*acting ncRNAs may act through transcriptional interference as has been postulated for the repression of the paternally imprinted *Igf2r* gene by the *Airn* ncRNA [91]. Alternatively, ncRNAs, such as *HOTTIP,* promote the local recruitment of the H3K4 trimethylase MLL to the *HOXA* gene cluster and together are thought to control *HOXA* gene activation [92]. In contrast, *trans-*acting ncRNAs may function as scaffolds for protein complexes. For example, *HOTAIR* is transcribed from the *HOXC* locus and complexes with the Polycomb Repressive Complex 2 (PRC2) and KDM1 demethylase in order to localize to and silence the *HOXD* locus in humans [93-95]. *Drosophila roX* RNAs work in combination with the Male Specific Lethal (MSL) complex to up-regulate expression of X-chromosome linked genes *in trans* during dosage compensation in male flies [96].

Non-coding RNAs can also influence mRNA processing, such as alternative splicing, for example, through the direct interaction of the *MALAT1* ncRNA with splicing factors [97]. Furthermore, the levels of splice isoforms for a number of mRNAs, such as the α-thyroid hormone receptor gene *erbAα,* correlate with the amount of overlapping antisense transcripts [81]. In this context it is interesting to note that alternative splicing has been linked to both nucleosomal organization in general and H3 K36 methylation in particular, both of which are affected by the process of antisense transcription. Exons are enriched for nucleosomes, and constitutively transcribed exons show particularly high levels of H3 K36me3 compared to alternative exons [98,99]. H3 K36 trimethylation is not the only histone modification to affect splicing. Hence, it will be interesting to determine the involvement of H3K36 methylation in histone dynamics and thus its impact on alternative splicing in higher eukaryotes and to determine to what extent the molecular mechanisms are conserved from yeast to man.

## Abbreviations

Airn: Antisense to Igf2r RNA non-coding; Asf: Anti-silencing factor; ChIP-Chip: Chromatin immunoprecipation on microarray; ChIP-seq: Chromatin immunoprecipitation sequencing; CTD: C-terminal domain; Ctk: C-terminal kinase; CUT: Cryptic unstable transcripts; Chd: Chromodomain-helicase-DNA binding; DNA: Deoxyribonucleic acid; Eaf: Essential SAS2-related Acetyltransferase 1 (ESA1) associated factor; FACT: Facilitates chromatin transcription; GAL: Galactose metabolism; H2A: Histone 2A; H2B: Histone 2B; H3: Histone 3; H4: Histone 4; Hda: Histone deacetylase; Hos,: Hda One Similar; HOTAIR: HOX antisense intergenic RNA; HOTTIP: HOXA distal tip transcript antisense RNA; HOX: Homeobox; Hst: Homolog of SIR2; IME: Inducer of meiosis; Isw: Imitation Switch; K: Lysine; KDAC: Lysine deacetylases; KDM: Lysine demethylase 1; KMT: Lysine methyl transferase; MALAT1: Metastasis-associated lung adenocarcinoma transcript 1; Me: Methylated; me3: Trimethylated; MLL: Mixed-lineage leukemia; MNase: Micrococcal nuclease; MSL: Male Specific Lethal; NDR: Nucleosome depleted region; Poly(dA:dT): Polymer of deoxy-adenylic and deoxy-thymidylic acids; PHD: Plant-Homeodomain; PHO: Phosphate metabolism; PRC2: Polycomb repressive complex 2; PWWP: Proline-tryptophan-tryptophan-proline motif; Rme1: Regulator of Meiosis 1; RNA: Ribonucleic acid; RNAPII: RNA Polymerase II; RNA-seq: Ribonucleic acid sequencing; mRNA: Messenger ribonucleic acid; ncRNA: Non-coding RNA; rRNA: Ribosomal ribonucleic acid; tRNA: Transfer ribonucleic acid; roX: RNA on the X; Rpb: RNA Polymerase B; Rpd3S: Reduced potassium dependency 3 (Rpd3) containing complex Small; RSC: Remodels Structure of Chromatin; Ser: Serine; SER: Serine requiring; Set: Suv39H E(z), Trithorax domain containing; Set3C: Set3-containing complex; Sin: Switch independent; Spt: Suppressor of Ty1 transposon; SRG: SER3 regulatory gene; SRI: Set2-Rpb1 interaction domain; SSU72: Suppressor of SUa7 gene 2; SUT: Stable unannotated transcript; TSS: Transcription start site; Ume: Unscheduled meiotic gene expression.

## Competing interests

The authors declare that they have no competing interests.

## Authors’ contributions

This work is supported by NIH grant R01GM047867 to JLW and the Stowers Institute for Medical Research.
